# Blood flow restriction training before platelet‐rich plasma preparation induces a significant reduction in its interleukin‐6 levels: A pilot randomised controlled trial

**DOI:** 10.1002/jeo2.70500

**Published:** 2025-10-30

**Authors:** Óscar Daniel Omaña Ávila, Rafael José Melo Cué, María Victoria Romero Rodríguez, Cassandra Pacheco, María Isabel Mijares, Fhabián Stevens Carrión‐Nessi, Olivia González Cordero, Stefano Zaffagnini, Theodorakys Marín Fermín

**Affiliations:** ^1^ Biomedical Research and Therapeutic Vaccines Institute (VACTER) Ciudad Bolivar Venezuela; ^2^ “Luis Razetti” School of Medicine Central University of Venezuela Caracas Venezuela; ^3^ Laboratorio Avilab Caracas Venezuela; ^4^ Clínica Santa Sofía Caracas Venezuela; ^5^ 2nd Orthopaedic and Trauma Department IRCCS Rizzoli Orthopaedic Institute Bologna Italy

**Keywords:** blood flow restriction, growth factors, insulin‐like growth factor 1, interleukin 6, knee extensions, orthobiologics, platelet‐rich plasma

## Abstract

**Purpose:**

To assess platelet‐rich plasma (PRP) changes in platelet and leucocyte count, insulin‐like growth factor (IGF‐1) and interleukin 6 (IL‐6) concentration after bilateral low‐load knee extensions with blood flow restriction (BFR).

**Methods:**

A randomised controlled trial involving two groups was conducted. The intervention group underwent low‐load bilateral knee extensions with BFR, while the control group without BFR. Inclusion criteria were (1) male individuals, (2) between 18 and 40 years, (3) with Tegner activity level ≥5 and (4) no musculoskeletal conditions that would interfere with exercise. The participant performed a standard protocol of 30–15–15–15 repetitions of consecutive sets with 30‐s rest intervals at 80% of limb occlusive pressure and a 15‐lb load. PRP platelet and leucocyte count, IGF‐1 and IL‐6 concentration measurements (via flow cytometry, chemiluminescence testing and immunochromatography, respectively) were conducted before exercise and 10, 20 and 30 min after the intervention (T1, T2, T3 and T4, respectively).

**Results:**

A total of 24 participants were enrolled and allocated into two groups. The exercise bout in the BFR group resulted in higher platelet concentration and dose at T2 and T3, reaching a mean 9.9% maximum concentration increase and a mean 15.6% maximum dose increase at T2 (*p* = 0.07). Similarly, leucocyte concentrations and dose presented a steep decrease at T2, representing a 17.8% and 16.8% decrease, respectively. Moreover, a peak increase in IGF‐1 concentration of 4.1% above baseline was observed at T3. IL‐6 concentrations were significantly lower at all time points in the BFR group (˂1 pg/mL, *p* = 0.01).

**Conclusion:**

Low‐load bilateral knee extensions with BFR induced a significant reduction of IL‐6 concentration in PRP, which holds potential to tailor treatment for specific musculoskeletal injuries.

**Level of Evidence:**

Level I.

**Abbreviations**
ANOVA: analysis of variance
BFR: blood flow restriction
IGF‐1: insulin‐like growth factor 1
IL‐6: interleukin 6
PRP: platelet‐rich plasma

## INTRODUCTION

Platelet‐rich plasma (PRP) is a worldwide implemented orthobiologics therapy that aims to enhance the regeneration potential of musculoskeletal tissues through its immunomodulatory properties [21]. Although its definition remains controversial, it is a plasma portion of blood that displays a higher concentration of platelets than baseline after centrifugation [29]. PRP has established itself as a valid conservative option for managing knee osteoarthritis [11, 16]. However, its uses span from cartilage to bone and from acute traumatic events to degenerative sports‐related injuries [19, 22, 24].

Over the last few decades, the role of PRP components has been widely studied [4, 23]. Platelet concentration and dose (defined as the absolute amount of platelets delivered per injection) are critical for the therapy's success [4, 9]. Similarly, PRP leucocyte concentration profiles—leucocyte‐poor or leucocyte‐rich—may yield improved benefits in specific conditions such as osteoarthritis or tendinopathies [1, 8, 12, 17, 35, 41].

Exercise effects on PRP have been found to modify the quantity and quality of its components [5, 6, 14]. In that sense, blood‐flow restriction (BFR) therapy is a fascinating exercise modality with local and systemic effects that can potentially change PRP composition [30]. BFR creates a venous stasis due to applying a tourniquet at partial occlusive pressures in the proximal limb while performing low‐load exercises [25, 32]. It promotes a metabolic stress environment that triggers the release of multiple hormones and growth factors, including increased insulin‐like growth factor 1 (IGF‐1) and interleukin 6 (IL‐6) concentration and generates muscle strength gains similar to traditional resistance training [15, 20, 28, 31, 33, 34, 39].

IGF‐1 and IL‐6 may play a significant role in the proliferation and maturation of chondrocytes, inhibiting the apoptosis of osteoarthritic chondrocytes and promoting muscle hypertrophy and regeneration, respectively [36, 38]. Although both IGF‐1 and IL‐6 have been shown to contribute to musculoskeletal tissue healing, the opportunity to treat muscle injuries is the most promising, as the evidence on PRP has shown poor outcomes [22, 26, 27, 37]. Given its safety profile and ease of use [18], BFR‐enhanced PRP can alter PRP composition in an office‐based setting with potential applications in sports‐related injuries.

The present study aims to assess PRP changes in platelet and leucocyte count, IGF‐1 and IL‐6 concentration after bilateral low‐load knee extensions with BFR. The hypothesis is that bilateral low‐load knee extensions with BFR will increase platelet and leucocyte counts, IGF‐1 and IL‐6 in PRP prepared after the exercise bout.

## METHODS

### Study design

A randomised controlled trial involving two groups was conducted at Laboratorio Avilab in Caracas, Venezuela, following the study protocol [3] and the Declaration of Helsinki. The study was approved by the Research and Bioethics Committee of Clínica Santa Sofía and prospectively registered under the BioMed Central‐International Standard Randomised Controlled Trial Number ISRCTN42221463. The intervention group underwent low‐load bilateral knee extensions with BFR, while the control group underwent low‐load bilateral knee extensions without BFR.

### Participants and eligibility criteria

Healthy volunteers were enrolled via public call in the study after meeting predefined criteria, following an interview to collect demographic and anthropometric data, and providing written informed consent. Inclusion criteria were (1) male individuals, (2) between 18 and 40 years, (3) with Tegner activity level ≥5 and (4) no musculoskeletal conditions that would interfere with exercise. Exclusion criteria included (1) individuals with systemic inflammatory diseases, (2) cardiovascular risk factors (including deep vein thrombosis, hypertension, lymphedema, history of endothelial dysfunction, varicose veins, peripheral vascular disease, active smoking), (3) any blood dyscrasia, (4) Tegner Activity scale scores <5, (5) under nonsteroidal anti‐inflammatory drugs and aspirin treatment within 1 week before testing or (6) that had previously performed exercises on the testing day. Participants were allowed to withdraw from the study at any time.

### Randomisation and allocation

Participants were block randomised into the intervention and control groups in a 1:1 ratio. The allocation sequence was generated using computer‐generated random numbers within blocks, ensuring balanced group sizes and minimising allocation bias.

### Outcome measures and assessment

Primary outcomes included PRP volume, platelet and leucocyte count and concentration, leucocyte differential analysis and IGF‐1 and IL‐6 concentration, which were assessed at baseline and 10, 20 and 30 min after the intervention. Each blood draw was performed by a single phlebotomist for each participant, and two certified bioanalysts independently handled the IGF‐1 and IL‐6 measurements. Additionally, adverse events were recorded in a logbook and promptly reported to the Research and Bioethics Committee. Follow‐up calls were made 72 h after blood sample collection to assess for the late presentation of any adverse event.

### Blood sample collection, PRP preparation and analysis

A multidisciplinary medical team led by the principal investigator conducted the intervention in an ISO 9001‐certified laboratory. First, participants underwent pre‐exercise peripheral vein catheterisation, blood sample draw and PRP preparation for baseline measurements at 21°C–22°C. Each participant underwent standard venipuncture in the antecubital fossa by a single phlebotomist under sterile conditions for a total blood draw of 15 mL, divided into three test tubes (Weihai Sunway Medical Technology Vacu‐T®): a 2.7 mL 3.2% sodium citrate blue tube and two 6 mL silica clot activator red tubes.

The 2.7 mL 3.2% sodium citrate blue tubes underwent a single centrifugation (Eppendorf 5702R) at 1500 rpm for 5 min (relative centrifugal force: 2900G). The plasma portion was separated from the red blood cells under direct visualisation with automatic pipettes, and samples were sent for analysis. The plasma was analysed with an Auto Haematology Analyzer with ESR (Mindray BC‐780R) for platelet, red cell and leucocyte counts, as well as leucocyte differential analysis using focusing flow‐DC impedance, optical and manual methods and SF cube fluorescent technology analysis.

The 6 mL silica clot activator red tubes underwent a single centrifugation (Eppendorf 5702R, Germany) at 3500 rpm for 15 min (Relative centrifugal force: 2900G). Approximately 3 mL of each tube was used for IGF‐1 and IL‐6 serum chemoluminescence, respectively. IGF‐1 chemiluminescence testing was conducted using a LIAISON Analyzer (DiaSorin). Serum samples were stored at −20°C, and IL‐6 analysis was performed in five‐sample batches using the iFlash 1800 (Shenzhen YHLO Biotech). The IL‐6 testing could only detect levels ˃1 pg/mL.

### Low‐load bilateral knee extensions with or without blood flow restriction protocol

**Figure 1:**
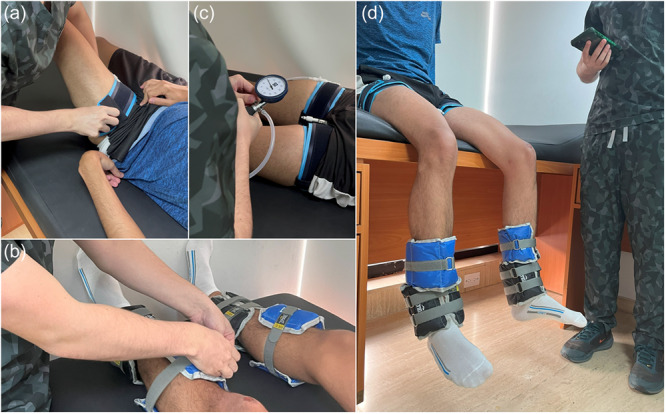
Low‐load bilateral knee extensions with blood flow restriction. (a) Tourniquet placement at the proximal end of both thighs; (b) Positioning of 15‐lb ankle weights; (c) Increasing cuff pressure to 80% of limb occlusive pressure; (d) The participant is sitting and ready to start the bilateral knee extensions.

The participant then performed a low‐load bilateral knee extension protocol with BFR (intervention group) (Figure 1) or without it (control group). The low‐load bilateral knee extensions under BFR, using tourniquets (The Occlusion Cuff Pro®) at the proximal end of both thighs, followed the standard protocol of four sets consisting of 30–15–15–15 repetitions, with 30‐s rest intervals at 80% of limb occlusive pressure (*arteria pedis*) and a total of 15 lbs in ankle weights. On the other hand, the control group performed the same bilateral knee extension protocol without the tourniquets. A staff physician monitored the entire exercise protocol and recovery period to ensure safety and collect data on adverse events. Once the exercise protocol was completed, participants were allowed a recovery period, which included rest, walking if desired, and a maximum of 5 oz of fluid intake, before undergoing the consecutive blood draws, performed identically to the first, at 10‐, 20‐ and 30‐min postintervention.

### Statistical analysis

A power analysis yielded a sample size of 22 patients, anticipating a 25% increase in IGF‐1 with a significance level (*α*) of 0.05 and 80% statistical power. Additionally, previous studies have been conducted using a similar sample size [5, 14, 34]. Patient data were summarised using the following descriptive statistics: mean and standard deviation, median and interquartile range and/or frequency and percentage (%). The distribution of numerical variables was assessed using the Shapiro–Wilk test. For numerical variables, the mixed analysis of variance (ANOVA) test was used for those with a normal distribution, and the Friedman test was used for those with a nonnormal distribution. Values of *p* < 0.05 were considered statistically significant. Statistical analysis was performed using SPSS version 27.

## RESULTS

**Figure 2:**
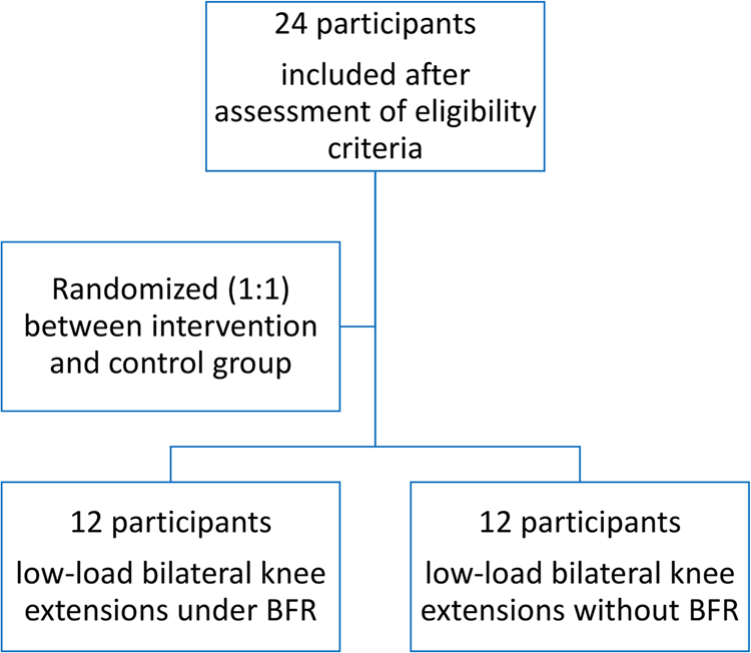
Blood flow restriction‐enhanced platelet‐rich plasma randomised controlled trial flowchart on participant recruitment, randomisation and group allocation.

**Figure 3:**
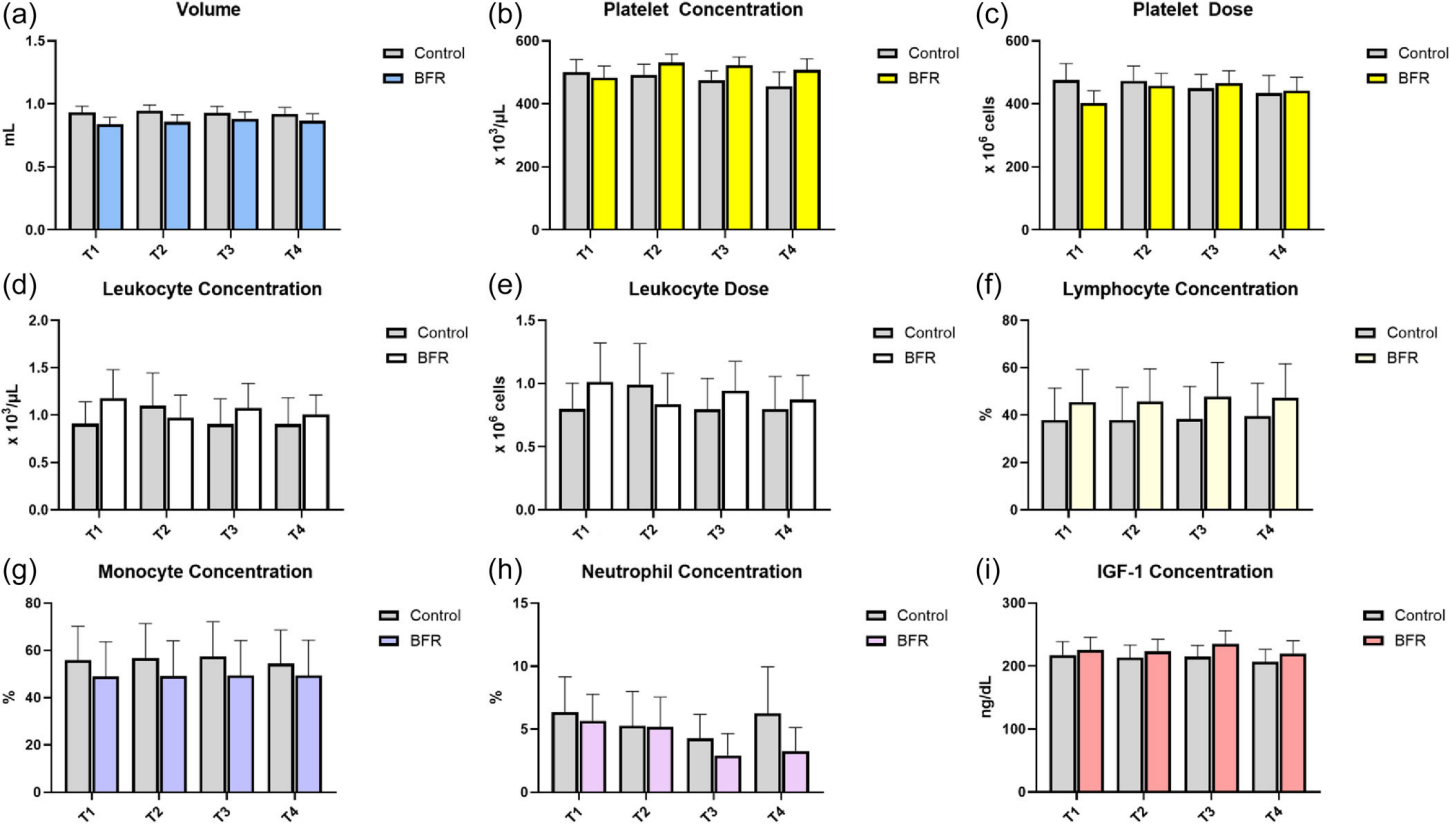
Platelet‐rich plasma composition means and standard error of the mean before and after low‐load bilateral knee extensions with and without blood flow restriction training in: (a) volume, (b) platelet concentration, (c) platelet dose, (d) leukocyte concentration, (e) leukocyte dose, (f) lymphocyte concentration, (g) monocyte concentration, (h) neutrophil concentration, and (i) insulin‐like growth factor 1. T1 = before exercise; T2 = 10 min after exercise; T3 = 20 min after exercise; and T4 = 30 min after exercise. BFR, blood flow restriction; IGF‐1, insulin‐like growth factor 1.

**Figure 4:**
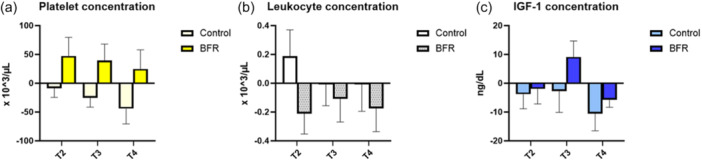
Mean differences from baseline and standard error of the mean of platelet, leucocyte and insulin‐like growth factor 1 concentration in platelet‐rich plasma after low‐load bilateral knee extensions with and without blood flow restriction training in: (a) platelet concentration, (b) leukocyte concentration, and (c) insulin‐like growth factor. Negative histograms mean that the concentration decreased below the baseline concentration. T2 = 10 min after exercise; T3 = 20 min after exercise; and T4 = 30 min after exercise. BFR, blood flow restriction; IGF‐1, insulin‐like growth factor 1.

From April to November 2024, 24 consecutive participants were enrolled and allocated into two groups (Figure 2). Participants' demographic and anthropometric data showed no significant differences (Table 1). The participants were males with a mean age of 25 ± 4 years, a height of 170 ± 10 cm, a weight of 71.7 ± 13.9 kg, a mean body mass index of 24.5 ± 4, and a Tegner activity level of 6.1 ± 1. Both groups showed PRP composition alterations after the exercise bout, yet no significant differences were found except for IL‐6 concentration (Tables 2 and 3, Figures 3 and 4).

**Table 1:** Participants' demographic and anthropometric data.

Variables	Control group	BFR group
Age (years)	25.3 ± 4.7	25.3 ± 4.4
Height (centimetres)	169 ± 0.01	173 ± 0.01
Weight (kilograms)	72.4 ± 17.3	70.9 ± 10.2
Body mass index	25.3 ± 5.1	23.6 ± 2.4
Tegner activity scale	6.3 ± 1.1	6.0 ± 0.9

Abbreviation: BFR, blood flow restriction training.

**Table 2:** Platelet‐rich plasma composition before and after low‐load bilateral knee extensions with and without blood flow restriction training.

Group	PRP volume (mL)	Platelet concentration (x10^3^/µL)	Platelet dose (x10^6^)	Leucocyte concentration (x10^3^/µL)	Leucocyte dose (x10^6^)	Lymphocytes (%)	Monocytes (%)	NEUTROPHILS (%)	IGF‐1 (ng/dL)
Control group									
T1	0.933 ± 0.17	501 ± 14	475.900 ± 180.75	0.911 ± 0.79	0.799 ± 0.070	38 ± 47	56 ± 50	6 ± 10	217.450 ± 74.05
T2	0.945 ± 0.16	492 ± 12	473.025 ± 164.18	1.10 ± 1.19	0.991 ± 1.13	38 ± 47	57 ± 50	5 ± 9	213.633 ± 68.46
T3	0.929 ± 0.18	475 ± 10	449.912 ± 150.60	0.903 ± 0.93	0.793 ± 0.85	38 ± 48	57 ± 51	4 ± 7	214.758 ± 62.43
T4	0.920 ± 0.18	456 ± 16	434.337 ± 195.76	0.903 ± 0.97	0.796 ± 0.88	39 ± 49	54 ± 50	6 ± 13	206.866 ± 67.90
BFR group									
T1	0.837 ± 0.20	483 ± 13	402.679 ± 133.06	1.180 ± 1.04	1.012 ± 1.07	45 ± 48	49 ± 51	6 ± 7	225.666 ± 69.76
T2	0.858 ± 0.19	531 ± 95	457.533 ± 136.49	0.97 ± 0.84	0.835 ± 0.85	46 ± 48	49 ± 51	5 ± 8	223.700 ± 65.96
T3	0.883 ± 0.18	523 ± 90	465.550 ± 136.92	1.073 ± 0.90	0.943 ± 0.81	48 ± 50	49 ± 52	3 ± 6	234.808 ± 73.69
T4	0.866 ± 0.19	508 ± 12	442.450 ± 145.82	1.005 ± 0.72	0.872 ± 0.67	47 ± 50	49 ± 52	3 ± 7	219.908 ± 69.68
Intragroup *p*‐value^a^	0.23^b^	0.16^b^	0.07^b^	0.34^c^	0.42^c^	0.78^b^	0.29^b^	0.78^c^	0.38^c^
Intergroup *p*‐value^a^	0.34	0.51	0.79	0.78	0.83	0.68	0.74	0.68	0.65

Abbreviations: BFR, blood flow restriction training; IGF‐1, insulin‐like growth factor 1; PRP, platelet‐rich plasma; T1, baseline values; T2, 10 min after intervention; T3, 20 min after intervention; T4, 30 min after intervention.

a Mixed analysis of variance. Intragroup *p*‐value is a general value for multiple comparisons of T1, T2, T3 and T4 within groups, and intergroup *p*‐value refers to control versus intervention group comparisons at the same timepoints (e.g., T1 control vs. T1 BFR group).

^b^ By Greenhouse–Geisser correction.

^c^ By Huynh–Feldt correction.

**Table 3:** Platelet‐rich plasma IL‐6 concentrations before and after low‐load bilateral knee extensions with and without blood flow restriction training.

Group	IL‐6^a^
Control group	
T1	5
T2	6
T3	4
T4	4
BFR group	
T1	2
T2	0
T3	1
T4	1
Intragroup *p*‐value^b^	0.216^c^
Intergroup *p*‐value^b^	0.013

*Note*: IL‐6 concentrations were significantly lower at all time points in the BFR group.

Abbreviations: BFR, blood flow restriction training; IL‐6, interleukin 6; T1, baseline values; T2, 10 min after intervention; T3, 20 min after intervention; T4, 30 min after intervention.

a Number of participants with IL‐6 levels ˃ 1 pg/mL.

b Mixed analysis of variance.

^c^ by Greenhouse–Geisser correction. Intragroup *p*‐value is a general value for multiple comparisons of T1, T2, T3 and T4 within groups, and intergroup *p*‐value refers to control versus intervention group comparisons at the same timepoints (e.g., T1 control vs. T1 BFR group).

The exercise bout in the control group yielded slight changes in the PRP composition except for a leucocyte concentration and dose peak increase at T2 (control leucocyte concentration T1 0.91 ± 0.79 × 10^3^/µL vs. T2 1.10 ± 1.19 × 10^3^/µL and control leucocyte dose T1 0.80 ± 0.70 × 10^6^ vs. T2 0.99 ± 1.13 × 10^6^), where a mean 20.88% and 23.75% increase to baseline was observed (Figures 3d,e and 4b).

On the other hand, the exercise bout in the BFR group resulted in higher platelet concentration and dose at T2 and T3, reaching a mean 9.94% maximum concentration increase and a mean 15.61% maximum dose increase at T2 (*p* = 0.07) (BFR platelet concentration T1 483 ± 128 × 10^3^/µL vs. T2 531 ± 95 × 10^3^/µL and T3 523 ± 90 × 10^3^/µL; BFR platelet dose T1 402.68 ± 133.06 × 10^6^ vs. T2 457.53 ± 136.49 × 10^6^ and T3 465.55 ± 136.92 × 10^6^). Similarly, leucocyte concentrations and dose presented a steep decrease at T2, representing a 17.80% and 16.83% decrease, respectively (BFR leucocyte concentration T1 1.18 ± 1.04 × 10^3^/µL vs. T2 0.97 ± 0.84 × 10^3^/µL and BFR leucocyte dose T1 1.01 ± 1.07 × 10^6^ vs. T2 0.84 ± 0.85 × 10^6^). Moreover, a peak increase in IGF‐1 concentration of 4.05% above baseline was observed at T3 (BFR IGF‐1 concentration T1 225.67 ± 69.76 ng/dL vs. 234.81 ± 73.69 ng/dL) (Figures 3b–e,i and 4).

Leucocyte differential analysis, which includes lymphocytes, monocytes and neutrophils, revealed no statistically significant differences (Figure 3f–h). However, IL‐6 concentrations were significantly lower at all time points in the BFR group (˂1 pg/mL, *p* = 0.013) (Table 3). Only one participant in the BFR group reported delayed onset of muscle soreness at 72 h postintervention; no other complication was noted among the participants.

## DISCUSSION

The main finding of the present study is that low‐load bilateral knee extensions with BFR induce a significant reduction of IL‐6 concentration in PRP, contrary to our hypothesis.

Previous studies have found that different exercise modalities modify the concentration of growth factors in PRP [5, 14]. In a controlled laboratory study by Hamilton et al. [14] comprising ten healthy individuals, they found significant suppression of vascular endothelial growth factor and platelet‐derived growth factor‐AB concentrations after an hour of submaximal cycling on an electronically braked cycle ergometer at 50% of peak power output. Similarly, Baria et al. [5], in their controlled laboratory study including ten healthy individuals, found a significant increase in transforming growth factor‐beta after a 4‐min high‐intensity interval cycling.

A previous laboratory study on the effects of BFR in PRP composition, conducted by Callanan et al. [6] on 16 participants, showed no differences in IL‐10, IL‐6, granulocyte‐macrophage colony‐stimulating factor, IL‐1ra, tumour necrosis factor‐α, or IL‐2 after 20 min of interval training in a recumbent cross‐trainer wearing a cooling vest set at a temperature of 8.3°C and BFR on the upper arm and upper leg at 40 and 65 mmHg. Contrary, our findings suggest a not statistically significant peak increase in IGF‐1 concentration of 4.05% above baseline 20 min after low‐load bilateral knee extensions with BFR (BFR IGF‐1 concentration T1 225.67 ± 69.76 ng/dL vs. 234.81 ± 73.69 ng/dL) and significantly lower IL‐6 concentrations at all time points (˂1 pg/mL, *p* = 0.013).

Likewise, previous research has assessed the effect of exercise in enhancing PRP platelet and leucocyte composition. Researchers agree that exercise can temporarily increase platelet concentration and dose [6, 14] to an even significant 23%–35% [2, 5], with similar increases in leucocytes and differential [2, 6]. In our study, although not statistically significant, low‐load bilateral knee extensions with and without BFR triggered different alterations, reaching in the BFR group a mean 9.94% maximum platelet concentration increase and a mean 15.61% maximum dose increase 10 min after the exercise bout but with a steep decrease of leucocyte concentration and dose of 17.80% and 16.83%, respectively. On the other hand, leucocyte concentration and dose peaked at 10 min (a mean 20.88% and 23.75% increase) in the control group.

These findings may suggest a trend that correlates the type and intensity of exercise and the individuals' fitness level with platelets and leucocyte response, as well as a potential role of the tourniquet cuffs in the mobilisation of the latter. The alteration of PRP composition induced by the implemented exercise bout with BFR can potentially alter the inflammatory and angiogenic pathways and interconnections mediated by IL‐6 signalling networks of PRP secretome that benefit musculoskeletal tissue healing [7, 13, 40, 43].

While IGF‐1 increased 20 min after BFR was not statistically significant, it can potentially hold a clinically relevant impact. An animal study by Zhang et al. [42] found that doses as low as 10 ng of IGF‐1, as those obtained in our pilot study, can produce more effective subchondral bone formation after full‐thickness articular cartilage repair of rabbits' knees. Furthermore, the IGF‐1 and IL‐6 changes elicited in our study synergistically align with reducing proinflammatory markers and mitigating cartilage and synovium damage in knee osteoarthritis and cartilage repair animal models [13, 40, 42, 43].

### Limitations

The limitations of the current study are inherent in its limited sample size and the specific patient characteristics, which hinder the extent of applicability. Additionally, the IL‐6 testing could only detect levels ˃1 pg/mL, restricting a deeper understanding of its behaviour during the experiment. Last, only male participants were recruited to avoid bias on the effect of the menstrual cycle on platelet function, hindering the translation of our findings in females [10]. Future studies should aim to find innovative ways to retrieve the systemic release of growth factors and hormones induced by BFR into PRP, assess its clinical effect, and develop tailored PRP compositions in treating specific musculoskeletal injuries.

## CONCLUSION

Low‐load bilateral knee extensions with BFR induced a significant reduction of IL‐6 concentration in PRP, which holds potential to tailor treatment for specific musculoskeletal injuries.

## AUTHOR CONTRIBUTIONS

**Óscar Daniel Omaña Ávila**: Formal analysis; investigation; data curation; writing—original draft; supervision. **Rafael José Melo Cué**: Investigation; supervision. **María Victoria Romero Rodríguez**: Investigation. **Cassandra Pacheco**: Investigation; resources. **María Isabel Mijares**: Investigation; resources. **Fhabián Stevens Carrión‐Nessi**: Validation; formal analysis; writing—review and editing; visualisation. **Olivia González Cordero**: Validation; investigation; writing—review and editing; supervision; project administration. **Stefano Zaffagnini**: Validation; writing—review and editing; supervision. **Theodorakys Marín Fermín**: Conceptualisation; methodology; validation; formal analysis; investigation; data curation; writing—original draft; writing—review and editing; supervision; project administration; funding acquisition.

## CONFLICT OF INTEREST STATEMENT

Theodorakys Marin Fermin reports financial support was provided by International Society of Arthroscopy Knee Surgery and Orthopaedic Sports Medicine. Stefano Zaffagnini and Theodorakys Marín Fermín are part of the *Journal of Experimental Orthopaedics* Editorial Board. The other authors declare no conflict of interest.

## ETHICS STATEMENT

All procedures performed in studies involving human participants were in accordance with the ethical standards of the institutional and/or national research committee and with the 1964 Helsinki Declaration and its later amendments or comparable ethical standards. This study was approved by the Research and Bioethics Committee of Grupo Médico Vargas‐Clínica Santa Sofía v.001‐2024 and prospectively registered under the BioMed Central‐International Standard Randomised Controlled Trial Number (ISRCTN42221463).

## Supporting information


**Checklist 1.** Consolidated Standards of Reporting Trials (CONSORT) statement checklist.

## Data Availability

The data underlying this article are available in the article and its online supplementary material.
